# Effectiveness of a helmet promotion campaign, China

**DOI:** 10.2471/BLT.22.287914

**Published:** 2022-03-23

**Authors:** Peishan Ning, Huiying Zong, Li Li, Peixia Cheng, David C Schwebel, Yang Yang, Lei Yang, Youyou Wu, Min Zhao, Guoqing Hu

**Affiliations:** a Department of Epidemiology and Health Statistics, Xiangya School of Public Health, Central South University, Changsha, 410078, China.; b Department of Psychology, University of Alabama at Birmingham, Birmingham, United States of America (USA).; c Department of Biostatistics, University of Florida, Gainesville, USA.

## Abstract

**Objective:**

To evaluate the effectiveness of a 2020 nationwide helmet promotion campaign, in terms of helmet wearing and correct helmet wearing, aimed at electric bike riders and motorcyclists in China.

**Methods:**

We obtained 192 hours of film of traffic before (2019) and after (2021) implementation of the campaign at eight road intersections in Changsha, recording cyclist (traditional and electric) and motorcyclist helmet-wearing behaviour during both weekdays and the weekend, and peak and off-peak traffic. We extracted data on rider characteristics and helmet-wearing behaviour. We applied a logistic regression to obtain estimates of helmet wearing and correct helmet wearing, and calculated odds ratios adjusted for rider variables.

**Findings:**

We filmed 11 525 cyclists and motorcyclists, 5256 (45.6%) before and 6269 (54.4%) after the campaign. We estimated a substantial increase in the overall percentage of helmet wearing from 8.8% (95% confidence interval, CI: 8.0–9.6) to 62.0% (95% CI: 60.8–63.2). After controlling for covariates, we noted that helmet wearing increased in all groups. However, we observed a decrease in the overall percentage of correct helmet wearing from 91.9% (95% CI: 89.4–94.3) to 83.5% (95% CI: 82.3–84.7). Post-campaign, we estimated the highest percentage of helmet wearing for delivery riders (88.8%) and lowest for traditional cyclists (3.8%); we estimated the lowest percentage of correct helmet wearing for three-wheeled motorcyclists (58.8%).

**Conclusion:**

To increase helmet wearing and correct helmet wearing, we recommend amending the campaign to include traditional cyclists as well as education and legislation on the correct fastening of helmet chinstraps.

## Résumé

**Objectif:**

Mesurer l'efficacité d'une campagne nationale organisée en 2020 afin de promouvoir l'adoption et le port adéquat du casque pour les motocyclistes et les cyclistes utilisant un vélo électrique en Chine.

**Méthodes:**

Nous avons obtenu 192 heures de vidéos provenant de caméras de surveillance routière placées sur huit carrefours à Changsha, avant (2019) et après (2021) la mise en œuvre de la campagne. Ces caméras ont filmé le comportement des motocyclistes ainsi que des cyclistes (sur vélo traditionnel et électrique) vis-à-vis du casque, en semaine et durant le week-end, mais aussi pendant les heures de pointe et les heures creuses. Nous y avons trouvé des informations sur les caractéristiques de ces usagers et leur attitude par rapport au casque. Enfin, nous avons appliqué un modèle de régression logistique en vue de formuler des estimations sur l'adoption et le port adéquat du casque, puis nous avons calculé l'odds ratio ajusté pour les variables relatives à ces usagers.

**Résultats:**

Nous avons filmé 11 525 cyclistes et motocyclistes, 5256 (45,6%) avant et 6269 (54,4%) après la campagne. Nous avons observé une importante augmentation du pourcentage total d'usagers portant un casque, de 8,8% (intervalle de confiance de 95%, IC: 8,0–9,6) à 62,0% (IC de 95%: 60,8-63,2). Après avoir pris en compte les covariables, nous avons remarqué que le port du casque avait augmenté dans toutes les catégories. Néanmoins, nous avons constaté une baisse du pourcentage total d'usagers portant correctement leur casque, de 91,9% (IC de 95%: 89,4-94,3) à 83,5% (IC de 95%: 82,3-84,7). À l'issue de la campagne, la catégorie d'usagers enregistrant le pourcentage le plus élevé de port du casque était celle des coursiers à vélo (88,8%), tandis que celle des cyclistes traditionnels affichait le plus faible pourcentage (3,8%). Nous avons également noté que les motocyclistes à trois roues constituaient la catégorie la moins bien représentée en matière de port adéquat (58,8%).

**Conclusion:**

Afin d'améliorer l'adoption et le port adéquat du casque, nous recommandons de modifier la campagne et d'y inclure les cyclistes traditionnels, mais aussi d'adapter la législation et l'éducation pour apprendre aux usagers à attacher correctement la mentonnière de leur casque.

## Resumen

**Objetivo:**

Evaluar la eficacia de una campaña de promoción del uso del casco a nivel nacional en 2020, en lo que respecta al uso del casco y a su correcta utilización, cuyo grupo objetivo son los conductores de bicicletas eléctricas y motociclistas en China.

**Métodos:**

Se obtuvieron 192 horas de filmación del tráfico antes (2019) y después (2021) de la aplicación de la campaña en ocho intersecciones viales de Changsha, en las que se registró el comportamiento de los ciclistas (con bicicletas tradicionales y eléctricas) y de los motociclistas en relación con el uso del casco, tanto en días laborables como en fin de semana, y en horas pico y no pico de tráfico. Se extrajeron datos sobre las características de los ciclistas y el uso del casco. Se aplicó una regresión logística para obtener estimaciones del uso del casco y de su correcto uso, y se calcularon las razones de posibilidades ajustadas a las variables de los conductores.

**Resultados:**

Se filmaron a 11 525 ciclistas y motociclistas, 5256 (45,6 %) antes y 6269 (54,4 %) después de la campaña. Se estimó un aumento sustancial del porcentaje global de uso del casco, que pasó del 8,8 % (intervalo de confianza del 95 %, IC: 8,0-9,6) al 62,0 % (IC del 95 %: 60,8-63,2). Tras controlar las covariables, se observó que el uso del casco aumentó en todos los grupos. No obstante, se observó una reducción en el porcentaje global de uso correcto del casco del 91,9 % (IC del 95 %: 89,4-94,3) al 83,5 % (IC del 95 %: 82,3-84,7). Después de la campaña, se estimó que el porcentaje más alto de uso del casco correspondía a los repartidores (88,8 %) y el más bajo a los ciclistas que utilizan bicicletas tradicionales (3,8 %); se estimó que el porcentaje más bajo de uso correcto del casco correspondía a los motociclistas con vehículos de tres ruedas (58,8 %).

**Conclusión:**

Para aumentar el uso del casco y su correcta utilización, se sugiere modificar la campaña para incluir a los ciclistas con bicicletas tradicionales, así como la educación y la legislación sobre la correcta sujeción de las correas de la barbilla del casco.

## ملخص

**الغرض:**

تقييم فعالية حملة الترويج لارتداء للخوذة على المستوى الوطني في عام 2020، من حيث ارتداء الخوذة والارتداء الصحيح للخوذة، والتي استهدفت راكبي الدراجات الكهربائية وراكبي الدراجات النارية في الصين.

**الطريقة:**

حصلنا على 192 ساعة من شريط تصوير لحركة المرور قبل تنفيذ الحملة (عام 2019)، وبعد تنفيذها (عام 2021) في ثمانية تقاطعات للطرق في تشانجشا، مع تسجيل سلوك راكبي الدراجات (التقليدية والكهربائية) وراكبي الدراجات النارية المرتدين للخوذة خلال أيام الأسبوع وعطلة نهاية الأسبوع، وأثناء ذروة حركة المرور، وغير أوقات الذروة. استخرجنا بيانات عن خصائص الراكب وسلوكه أثناء ارتداء الخوذة. قمنا بتطبيق انحدارًا لوجستيًا للحصول على تقديرات لارتداء الخوذة، والارتداء الصحيح للخوذة، مع حساب نسب الأرجحية المعدلة لمتغيرات الراكب.

**النتائج:**

قمنا بتصوير 11525 راكبًا للدراجات التقليدية والنارية، منهم 5256 (45.6%) قبل الحملة، و6269 (54.4%) بعد الحملة. قمنا بتقدير زيادة هائلة في النسبة المئوية الإجمالية لارتداء الخوذة من 8.8% (بفاصل ثقة مقداره 95%: 8.0 إلى 9.6) إلى 62.0% (بفاصل ثقة مقداره 95%: 60.8 إلى 63.2). بعد التحكم في المتغيرات المشتركة، لاحظنا أن ارتداء الخوذة قد ازداد في كل المجموعات. مع ذلك، لاحظنا انخفاضًا في النسبة المئوية الإجمالية لارتداء الخوذة بطريقة صحيحة من 91.9% (بفاصل ثقة مقداره 95%: 89.4 إلى 94.3) إلى 83.5% (بفاصل ثقة مقداره 95%: 82.3 إلى 84.7). بعد الحملة، قمنا بتقدير أعلى نسبة مئوية لارتداء الخوذة لدى راكبي توصيل الطلبات (88.8%)، وأدنى نسبة مئوية لراكبي الدراجات التقليدية (3.8%)؛ وكذلك تقدير أقل نسبة مئوية للارتداء الصحيح للخوذة لراكبي الدراجات النارية ذات العجلات الثلاث (58.8%).

**الاستنتاج:**

لزيادة ارتداء الخوذة والارتداء الصحيح للخوذة، فنحن نوصي بتعديل الحملة لتشمل راكبي الدراجات التقليدية، فضلًا عن التوعية والتشريع الخاص بالتثبيت الصحيح لأشرطة الخوذة على الرأس.

## 摘要

**目的:**

评估 2020 年全国头盔推广活动的效果，包括头盔佩戴和正确佩戴头盔情况，针对人群为中国电动车骑行者和摩托车骑行者。

**方法:**

我们获取了长沙市 8 个路口（2019 年）在该推广活动实施之前和（2021 年）之后 192 个小时的交通录像，录像记录了骑行者（传统自行车和电动车）和摩托车骑行者在工作日和周末、高峰和非高峰时段的头盔佩戴情况。我们提取了有关骑行者特征和头盔佩戴行为的数据。我们应用逻辑回归来获得头盔佩戴和正确佩戴头盔的估计值，并计算根据骑手变量调整的优势比。

**结果:**

我们拍摄了 11,525 名自行车和摩托车骑行者，其中活动之前 5,256 (45.6%) 人以及活动之后 6269 (54.4%) 人。我们估计头盔佩戴的总体百分比从 8.8% （95% 置信区间，CI：8.0–9.6）大幅增加到 62.0%（95% CI: 60.8–63.2）。在控制协变量后，我们注意到所有组的头盔佩戴率都有所增加。但是，我们也注意到正确佩戴头盔的总体百分比从 91.9%（95% CI: 89.4-94.3）下降至 83.5%（95% CI: 82.3-84.7）。活动结束后，我们估计快递骑手佩戴头盔的比例最高（88.8%），而传统骑行者佩戴头盔的比例最低（3.8%）；我们估计三轮摩托车骑行者正确佩戴头盔的比例最低（58.8%）。

**结论:**

为了增加头盔佩戴和正确佩戴头盔，我们建议修改活动纳入传统骑行者，以及教育和制定有关正确固定头盔下颏带的法规。

## Резюме

**Цель:**

Оценить эффективность общенациональной кампании 2020 года по поощрению использования шлемов с точки зрения их использования и правильного ношения водителями электровелосипедов и мотоциклистами в Китае.

**Методы:**

Авторы получили 192 часа видеозаписи дорожного движения до (2019 г.) и после (2021 г.) реализации кампании на восьми перекрестках дорог в городском округе Чанша, на которой отображено поведение велосипедистов (на обычных и электрических велосипедах) и мотоциклистов в шлемах в будние и выходные дни как в часы пик, так и вне часов пик. Авторы извлекли данные о характеристике водителей и их поведении в отношении использования шлема. Авторы применили логистическую регрессию для получения оценки использования шлемов и правильного их ношения, а также рассчитали отношение шансов с поправкой на переменные водителя.

**Результаты:**

Авторы засняли 11 525 велосипедистов и мотоциклистов, из них 5256 (45,6%) до и 6269 (54,4%) после кампании. Авторы оценили существенное увеличение общего процента водителей, которые использовали шлем, с 8,8% (95%-й ДИ: 8,0–9,6) до 62% (95%-й ДИ: 60,8–63,2). После учета ковариат авторы отметили, что использование шлема увеличилось во всех группах. Однако авторы наблюдали снижение общего процента водителей, которые использовали шлем правильно, с 91,9% (95%-й ДИ: 89,4–94,3) до 83,5% (95%-й ДИ: 82,3–84,7). После проведения кампании авторы оценили, что самый высокий процент водителей, которые использовали шлем, наблюдался среди водителей доставки (88,8%), а самый низкий – у обычных велосипедистов (3,8%). Авторы оценили, что самый низкий процент водителей, которые использовали шлем правильно, имел место среди водителей трехколесных мотоциклов (58,8%).

**Вывод:**

Чтобы увеличить количество водителей, которые используют шлем и носят его правильным образом, авторы рекомендуют внести изменения в кампанию, включив в нее обычных велосипедистов, а также использовать просвещение и законодательные акты о правильном креплении подбородочных ремней шлемов.

## Introduction

Cyclists and motorcyclists are vulnerable road users; of the 1 198 289 global road traffic deaths in 2019, cyclists and motorcyclists accounted for 64 693 (5.4%) and 222 357 (18.6%), respectively.^1^ The corresponding figures in China, the world’s most populous country, are 22 602 cyclist and 40 735 motorcyclist fatalities, contributing to 34.9% and 18.3% of global fatalities, respectively.^1^ These statistics are particularly remarkable given the recent achievements in other global health indicators such as life expectancy and maternal and child mortality.^2^

Helmet use reduces injuries and deaths among cyclists and motorcyclists,^3^^,^^4^ and has therefore become a recommended intervention in the World Health Organization (WHO) and United Nations (UN) *Global plan: decade of action for road safety 2021–2030*.^5^ This report, as well as previous research, also recommends the enactment of strong law enforcement on helmet requirements.^6^^–^^9^ Unfortunately, in 2017 just 49 of 167 countries that legally mandate motorcyclist helmets met five best-practice recommendations for helmet-use policies, and only 61 countries were rated as “good” in their helmet-wearing law enforcement.^6^

The estimated overall percentage of motorcyclists wearing a helmet was only 61.3% in low- and middle-income countries, much lower than the percentage of 94.4% in high-income countries.^6^ China, a middle-income country, implemented a national motorcyclist helmet law in 2003,^10^ but the percentage of motorcyclists wearing a helmet was only 20% in 2010.^6^ The 2003 law does not apply to cyclists (which includes riders of both traditional cycles and electric bikes, i.e. e-bikes) and is not strictly enforced for motorcyclists. According to a WHO/UN report, China only scored six points out of 10 for motorcyclist helmet law enforcement, with a score of 10 indicating regular and consistent enforcement.^6^

To address several road safety issues, including the low numbers of both e-bike riders and motorcyclists wearing a helmet, the Chinese government initiated the nationwide road safety campaign *One helmet, one belt*^11^ on 1 June 2020. The helmet promotion part of the campaign incorporated several different approaches, aiming to: increase helmet ownership, accessibility and utilization; strengthen the enforcement of existing laws and regulations; enhance national education; and implement on-site motorist counselling programmes to encourage increased helmet use (Box 1).^13^ The comprehensive campaign integrated many elements of empirically supported interventions.

Box 1 **Multifaceted approach of the 2020 national helmet promotion campaign targeted at e-bike riders and motorcyclists, China**

**1. Increasing helmet ownership, accessibility and utilization**

Three major processes took place: (i) 1.1 million helmets were donated by companies nationwide through more than 15 000 public events;^11^ (ii) over 75 000 public-access helmets were provided at traffic safety counselling points around schools and in rural areas before March 2021;^11^ and (iii) over 50 000 public-access e-bikes equipped with smart helmets that must be worn before the e-bike is unlocked were put into use in May 2021 in Changsha, China.^12^

**2. Strengthening law enforcement**

The current national law only mandates motorcyclists to wear a helmet, but the national helmet promotion campaign targeted both e-bike riders and motorcyclists (but not cyclists). The law enforcement component was led by the Traffic Management Bureau, who organized more than 3000 live streaming events and podcasts in which helmet law was enforced, and over 1000 coordinated and publicized (with reporters invited to participate) field enforcements of helmet-use laws and regulations. The bureau educated 12.2 million e-bike riders and motorcyclists violating helmet laws, and exposed typical cases of non-compliance.^11^

**3. Enhancing national education with regards to helmet use**

The education component included both offline (e.g. safety posters) and online (e.g. videos, audio, pictures and articles) methods to inform the public of helmet use, correct helmet use, helmet use as legislative or policy requirements, the enforcement plan for helmet use and fines for non-compliance. According to official statistics, over 3000 posters and 1000 educational videos had been produced to promote helmet use by March 2021. Further, over 300 000 educational messages and 20 000 videos had been disseminated through social media (e.g. WeChat and Microblog) and news and short film platforms. These efforts yielded 970 million Microblog readings and 10 billion short film plays.^11^

**4. Implementing on-site motorist counselling programmes**

On-site motorist counselling programmes, particularly focused on delivery riders, were conducted to persuade e-bike riders and motorcyclists to wear helmets. From May to November 2020, over 6100 on-site motorist counselling programmes were jointly implemented by 1.9 million traffic police departments and catering and express delivery industries across the country. These efforts resulted in over 630 000 delivery riders changing their helmet-wearing behaviour, and over 2300 police warnings were issued to catering and express delivery companies whose employees were not wearing helmets.^11^

We investigate whether the 2020 helmet-use promotion campaign was effective in increasing both helmet use and correct helmet use. Leveraging data collected for other purposes in 2019 in Changsha, China^14^ about one year before the initiation of the campaign, we compared pre-campaign observations with post-campaign data collected during the same season in 2021, about one year after the implementation of the campaign. Although the campaign was aimed at e-bike riders and motorcyclists, we also collected and analysed data concerning traditional cyclists. We converted these observed data from Changsha to estimated percentages of both helmet wearing and correct helmet wearing using a logistic regression model. 

## Methods

### Study design

We designed a film-based before-and-after study to evaluate the effectiveness of the national helmet promotion campaign. The pre-campaign observations were conducted from 29 June to 21 July 2019, and the post-campaign observations during 2–14 July 2021. We adopted multistage random sampling to determine observation sites (intersections) in Changsha, China.^15^ We recorded field observations using smartphone-based high-definition cameras (1080p resolution, Redmi Note 3, Xiaomi, Beijing, China) placed at each intersection to record all passing cyclists and motorcyclists.^15^

Because cyclist and motorcyclist behaviour (i.e. adherence to road safety regulations) varies between weekdays and weekends,^16^ and because law enforcement is inconsistent between weekdays and weekends, we recorded traffic at each selected intersection over two different days: one during the week and one during the weekend. On each recording day we filmed traffic for 6 hours: 3 hours of peak traffic and 3 hours of off-peak traffic. We determined the times of peak traffic through pilot observation.^15^ In total, we recorded traffic for 192 hours: two observations (pre- and post-campaign) at eight intersections/observation for 2 days/intersection (weekday and weekend) for 6 hours/day.

### Rider variables

Following techniques described in previous research,^17^^,^^18^ we transcribed the 192 hours of film to extract the following variables for each rider: (i) sex (male or female); (ii) estimated age group (< 20, 20–49 or ≥ 50 years); (iii) time of week (weekday or weekend); (iv) time of day (peak or off-peak traffic); (v) road user (traditional cyclist, e-bike rider, two-wheeled motorcyclist or three-wheeled motorcyclist); (vi) ownership type (public-access or privately owned vehicle), identified by viewer observation of the vehicle; and (vii) occupation (delivery rider or other), determined by viewer observation of the vehicle and/or uniform or apparel of rider. 

### Sample size

Because the 2019 pre-campaign observations to address other study hypotheses had already been conducted, we calculated the minimum sample size for this study based on study-specific aims and assumptions to assess whether the current sample size met those requirements. Because of potential clustering of riding behaviours at road intersections, we took intracluster correlation into account. Based on data from the 2019 study and relevant literature,^19^ assuming *α*: 0.05 and *β*: 0.80 (i.e. type I and II error values), helmet-wearing percentages pre- and post-campaign of 8.8% and 13.2%, respectively (i.e. a hypothesized 50% increase in helmet wearing), a cluster size of 650 and an intracluster correlation of 0.005, we determined the minimum sample size to be 3347 for each round of observations.

### Outcome measures

The primary outcome was the percentage of riders wearing a helmet, that is, the number of both cyclists and motorcyclists wearing a helmet as a percentage of the total number of filmed cyclists and motorcyclists. The secondary outcome was percentage of helmet-wearing riders correctly wearing a helmet. Following previous research, helmet wearing was recorded as correct if the helmet chinstrap was observed to be firmly fastened under the rider’s chin.^3^^,^^5^^,^^17^^,^^18^

### Statistical analysis

We used *χ^2^* tests to examine sample characteristic differences between pre- and post-campaign observations. We estimated the percentages of helmet wearing and correct helmet wearing and constructed 95% confidence intervals (CIs) assuming binomial distributions. Because intracluster correlations for percentages of helmet wearing (0.0786) and correct helmet wearing (0.0442) based on road intersection were both insignificant (*P*-values of 0.07 and 0.11, respectively), we tested the effectiveness of the national campaign using a logistic regression. We quantified the effect of the campaign on helmet wearing by estimating crude odds ratios (cORs) as well as adjusted odds ratios (aORs), adjusted for the seven rider variables described above. We performed all statistical analyses using Statistical Analysis System version 9.4 (SAS Institute, Cary, United States of America). Statistical tests were two-sided (i.e. the outcome variable differed across both the pre- and post-campaign observations) and conducted at *P* < 0.05.

### Ethical statement

The Ethics Committee of Xiangya School of Public Health, Central South University, China approved our study (approval no. XYGW-2021–72), exempting our research from informed consent. All our recorded data were de-identified. This report follows the Strengthening the Reporting of Observational Studies in Epidemiology statement.^20^

## Results

We recorded the helmet-wearing behaviour of 11 525 cyclists and motorcyclists in total. Both the 5256 (45.6%) recorded before and 6269 (54.4%) recorded after implementation of the national helmet promotion campaign exceeded our calculated minimum sample size required to estimate the percentage of riders wearing a helmet and conduct the desired statistical comparison (Table 1).

**Table 1:** Characteristics of filmed cyclists and motorcyclists before and after 2020 implementation of national helmet promotion campaign, Changsha, China

Variable	No. (%)		
	Total (*n* = 11 525)	Pre-campaign (29 June–21 July 2019) (*n* = 5 256)	Post-campaign (2–14 July 2021) (*n* = 6 269)
**Sex (*P* < 0.05)**			
Male	9 047 (78.5)	4 242 (80.7)	4 805 (76.6)
Female	2 478 (21.5)	1 014 (19.3)	1 464 (23.4)
**Age group, years (*P* < 0.05)**			
< 20	174 (1.5)	104 (2.0)	70 (1.1)
20–49	10 621 (92.2)	4 710 (89.6)	5 911 (94.3)
≥ 50	730 (6.3)	442 (8.4)	288 (4.6)
**Time of week (*P* < 0.05)**			
Weekday	4 869 (42.2)	2 013 (38.3)	2 856 (45.6)
Weekend	6 656 (57.8)	3 243 (61.7)	3 413 (54.4)
**Time of day (*P* = 0.20)**			
Peak	6 162 (53.5)	2 851 (54.2)	3 311 (52.8)
Off-peak	5 363 (46.5)	2 405 (45.8)	2 958 (47.2)
**Road user (*P* < 0.05)**			
Traditional cyclist	1 440 (12.5)	939 (17.9)	501 (8.0)
Public-access	1 004 (8.7)	687 (13.1)	317 (5.1)
Privately owned	436 (3.8)	252 (4.8)	184 (2.9)
E-bike rider	2 777 (24.1)	909 (17.3)	1 868 (29.8)
Public-access	1 394 (12.1)	151 (2.9)	1 243 (19.8)
Privately owned	1 383 (12.0)	758 (14.4)	625 (10.0)
Two-wheeled motorcyclist	7 035 (61.0)	3 246 (61.8)	3 789 (60.4)
Public-access	46 (0.4)	5 (0.1)	41 (0.7)
Privately owned	6 989 (60.6)	3 241 (61.7)	3 748 (59.7)
Three-wheeled motorcyclist^a^	273 (2.4)	162 (3.1)	111 (1.8)
**Occupation**			
Delivery rider	1 475 (12.8)	591 (11.2)	884 (14.1)
Other	10 050 (87.2)	4 665 (88.8)	5 385 (85.9)

E-bike: electric bike.

^a^ All three-wheeled motorcyclists were riding privately owned vehicles.

Between the two rounds of observation, we noted that the proportion of four types of riders (as a percentage of total number of riders) increased significantly, consistent with national trends: female riders increased from 19.3% (1014/5256) to 23.4% (1464/6269); riders aged 20–49 years increased from 89.6% (4710/5256) to 94.3% (5911/6269); riders of public-access e-bikes increased from 2.9% (151/5256) to 19.8% (1243/6269); and the percentage of delivery riders increased from 11.2% (591/5256) to 14.1% (884/6269; Table 1).

We estimated an increase in the overall percentage of all cyclists and motorcyclists wearing a helmet from 8.8% (95% CI: 8.0–9.6) to 62.0% (95% CI: 60.8–63.2) as a result of the national road safety campaign (aOR: 27.7; 95% CI: 24.5–31.3; Table 2). After controlling for covariates, we estimated that the helmet-wearing percentage of all groups increased substantially following the implementation of the campaign, with aORs ranging from 4.4 (95% CI: 1.2–16.4) for riders aged < 20 years to 54.3 (95% CI: 13.3–222.0) for riders of public-access vehicles.

**Table 2:** Estimated percentage of cyclists and motorcyclists wearing a helmet before and after implementation of 2020 national helmet promotion campaign, China

Variable	Percentage wearing a helmet (95% CI)			OR (95% CI)	
	Pre-campaign (29 Jun–21 Jul 2019)	Post-campaign (2–14 Jul 2021)		Crude OR	Adjusted OR^a^
**Overall**	8.8 (8.0–9.6)	62.0 (60.8–63.2)		16.9 (15.2–18.8)	27.7 (24.5–31.3)
**Sex**					
Male	9.5 (8.6–10.3)	65.6 (64.3–67.0)		18.2 (16.2–20.5)	28.5 (24.9–32.6)
Female	6.0 (4.6–7.5)	50.2 (47.6–52.8)		15.8 (12.0–20.9)	25.0 (18.4–33.8)
**Age group, years**					
< 20	3.9 (0.2–7.5)	17.1 (8.3–26.0)		5.2 (1.6–16.8)	4.4 (1.2–16.4)
20–49	9.0 (8.1–9.8)	62.9 (61.7–64.2)		17.2 (15.3–19.2)	28.2 (24.8–32.0)
≥ 50	8.1 (5.6–10.7)	54.5 (48.8–60.3)		13.5 (8.9–20.4)	31.3 (18.6–52.4)
**Time of week **					
Weekday	7.3 (6.2–8.4)	59.9 (58.2–61.7)		18.6 (15.5–22.3)	30.8 (25.0–37.9)
Weekend	9.7 (8.7–10.7)	63.8 (62.2–65.4)		16.4 (14.4–18.8)	26.3 (22.5–30.7)
**Time of day**					
Peak	8.1 (7.1–9.1)	61.0 (59.3–62.6)		17.9 (15.4–20.9)	29.9 (25.2–35.5)
Off-peak	9.7 (8.5–10.8)	63.2 (61.5–64.9)		15.8 (13.6–18.5)	25.4 (21.3–30.3)
**Road user**					
Traditional cyclist	0.8 (0.2–1.3)	3.8 (2.1–5.5)		5.2 (2.2–12.6)	5.2 (2.1–12.7)
E-bike rider	8.1 (6.4–9.9)	45.7 (43.5–48.0)		9.2 (7.1–11.8)	24.0 (17.6–32.7)
Two-wheeled motorcyclist	11.6 (10.5–12.7)	78.7 (77.4–80.0)		28.3 (24.8–32.3)	30.3 (26.4–34.8)
Three-wheeled motorcyclist	2.5 (0.1–4.9)	30.6 (22.1–39.2)		13.9 (5.2–36.8)	13.8 (5.1–37.4)
**Ownership type**					
Public-access	0.2 (0.0–0.6)	28.5 (26.3–30.8)		168.5 (41.9–677.6)	54.3 (13.3–222.0)
Privately owned	10.4 (9.5–11.3)	73.5 (72.2–74.8)		23.8 (21.2–26.7)	27.6 (24.4–31.3)
**Occupation**					
Delivery rider	18.4 (15.3–21.6)	88.8 (86.7–90.9)		35.9 (26.7–48.2)	47.8 (34.1–67.1)
Other	7.6 (6.8–8.3)	57.6 (56.3–59.0)		16.5 (14.6–18.6)	25.7 (22.5–29.3)

CI: confidence interval; E-bike: electric bike; OR: odds ratio.

^a^ Odds ratio after adjusting for seven covariates (sex, age group, time of week, time of day, type of road user, public-access versus privately owned vehicle and occupation) for overall helmet-wearing percentage, and after adjusting for the remaining six covariates for subgroup helmet-wearing percentage.

Notably, large post-campaign differences exist between groups (Table 2). For example, we estimated that 88.8% (95% CI: 86.7–90.9) of delivery riders were wearing helmets after the road safety campaign, compared with only 3.8% (95% CI: 2.1–5.5) of traditional cyclists. We also estimated low percentages of helmet wearers for riders younger than 20 years (17.1%; 95% CI: 8.3–26.0) and for riders of public-access vehicles (28.5%; 95% CI: 26.3–30.8).

Of cyclists and motorcyclists wearing helmets, we estimated correct helmet wearing in > 70% of all groups at both observation periods, with the exception of three-wheeled motorcyclists after implementation of the campaign (58.8%; 95% CI: 42.3–75.4; Table 3). However, we estimated a decrease in the overall percentage of correct helmet wearing from 91.9% (95% CI: 89.4–94.3) to 83.5% (95% CI: 82.3–84.7) between the two observation rounds, with an aOR of 0.5 (95% CI: 0.3–0.7). We also estimated a significant reduction in correct helmet wearing in most groups, with aORs as low as 0.2 (weekend riders or three-wheeled motorcyclists) to 0.5 (male, riders of privately owned vehicles or non-delivery riders). The decrease in the percentage of other helmet-wearing groups who were wearing helmets correctly was not significant. Of all groups, we estimated that the percentage of helmet wearers wearing helmets correctly was highest among delivery riders after the implementation of the campaign (90.3%; 95% CI: 88.3–92.4; Table 3). 

**Table 3:** Estimated percentage of helmet-wearing cyclists and motorcyclists wearing a helmet correctly before and after implementation of 2020 national helmet promotion campaign, China

Variable	Percentage of helmet wearers wearing helmet correctly (95% CI)			OR (95% CI)	
	Pre-campaign (29 Jun–21 Jul 2019)	Post-campaign (2–14 Jul 2021)		Crude OR	Adjusted OR^a^
**Overall**	91.9 (89.4–94.3)	83.5 (82.3–84.7)		0.4 (0.3–0.6)	0.5 (0.3–0.7)
**Sex**					
Male	91.4 (88.6–94.1)	81.7 (80.3–83.0)		0.4 (0.3–0.6)	0.5 (0.3–0.7)
Female	95.1 (89.7–100.0)	91.2 (89.1–93.2)		0.5 (0.2–1.8)	0.6 (0.2–2.1)
**Age group, years**					
< 20	100.0 (39.8–100.0)	83.3 (62.3–100.0)		NA^b^	NA^b^
20–49	93.0 (90.5–95.4)	83.8 (82.6–84.9)		0.4 (0.3–0.6)	0.4 (0.3–0.6)
≥ 50	77.8 (64.2–91.4)	77.1 (70.5–83.7)		1.0 (0.4–2.3)	1.0 (0.4–2.6)
**Time of week**					
Weekday	84.1 (78.3–89.9)	83.5 (81.7–85.2)		1.0 (0.6–1.5)	0.9 (0.6–1.5)
Weekend	95.6 (93.3–97.8)	83.5 (82.0–85.1)		0.2 (0.1–0.4)	0.2 (0.1–0.4)
**Time of day**					
Peak	91.7 (88.2–95.3)	83.3 (81.7–84.9)		0.4 (0.3–0.7)	0.5 (0.3–0.8)
Off-peak	92.0 (88.5–95.4)	83.7 (82.0–85.4)		0.4 (0.3–0.7)	0.5 (0.3–0.8)
**Road user**					
Traditional cyclist	100.0 (59.0–100.0)	73.7 (53.9–93.5)		NA^b^	NA^b^
E-bike rider	87.0 (79.5–94.5)	76.4 (73.6–79.2)		0.5 (0.2–1.0)	0.6 (0.3–1.3)
Two-wheeled motorcyclist	92.8 (90.2–95.4)	85.9 (84.6–87.1)		0.5 (0.3–0.7)	0.4 (0.3–0.7)
Three-wheeled motorcyclist	80.0 (44.9–100.0)	58.8 (42.3–75.4)		0.4 (0.0–3.5)	0.2 (0.0–2.8)
**Ownership type**					
Public-access	100.0 (15.8–100.0)	70.7 (66.6–74.9)		NA^b^	NA^b^
Privately owned	91.8 (89.3–94.3)	85.2 (84.0–86.4)		0.5 (0.4–0.7)	0.5 (0.3–0.7)
**Occupation**					
Delivery rider	96.3 (92.8–99.9)	90.3 (88.3–92.4)		0.4 (0.1–1.0)	0.4 (0.1–1.0)
Other	90.5 (87.4–93.5)	81.8 (80.4–83.1)		0.5 (0.3–0.7)	0.5 (0.3–0.7)

CI: confidence interval; E-bike: electric bike; NA: not applicable; OR: odds ratio.

^a^ Odds ratio after adjusting for seven covariates (sex, age group, time of week, time of day, type of road user, public-access versus privately owned vehicle and occupation) for overall percentage of correct helmet wearing, and after adjusting for the remaining six covariates for subgroup percentage of correct helmet wearing.

^b^ Crude and adjusted odds ratios were not calculated when one percentage for comparison is 0% or 100%.

## Discussion

The sixfold increase in helmet wearing after implementation of the national road safety campaign suggests that it was a highly successful intervention. Although the effectiveness of the campaign may be diminished in rural areas and smaller cities, where relevant resources are reduced compared with larger cities,^3^ we hypothesize that the overall success of the campaign was the result of several factors. First, the comprehensive, theory-driven, socioculturally tailored campaign matched recommendations for effective public health prevention programmes, and was widely implemented by a competent intervention team.^21^^,^^22^ Second, the goals of the campaign were strictly enforced by the local road traffic department, who engaged with the public to ensure helmet use. For example, over 1400 on-site enforcement campaigns were conducted by 7768 police officers between April and June 2021 in the two districts of Changsha, reprimanding 31 163 e-bike riders for riding without a helmet.^23^^,^^24^ We found no evidence of on-site enforcement conducted before the campaign. Third, awareness of the campaign among the public was high; an unpublished survey conducted by our team in August 2021 of 312 e-bike riders and motorcyclists revealed that 289 (92.6%) respondents had some knowledge of the national road safety campaign.^15^

The variation in percentages of riders wearing helmets between groups reflects the priorities of the national helmet promotion campaign: the intervention was targeted at all e-bike riders and motorcyclists, but not traditional cyclists.^13^ This inconsistency likely caused confusion among both traditional cyclists and traffic police officers. Governmental efforts to increase use of helmets by riders of public-access vehicles were also an important contributor to the inconsistent success of the campaign. The first set of 50 000 public-access e-bikes equipped with smart helmets were installed in Changsha in May 2021;^12^ these e-bikes have built-in fourth-generation (4G) technological communication components that create a wireless internet link between the smart helmet and e-bike, and the e-bike will not unlock for use if the smart helmet is not worn.^12^^,^^25^ Similar programmes were not introduced for public-access bicycles or motorcycles. Finally, the on-site motorist counselling programmes focused primarily on helmet use among delivery riders,^11^^,^^13^^,^^26^ contributing to the high post-campaign percentage of helmet wearers among this population.

In contrast, we estimated that the overall post-campaign percentage of helmet wearers who were wearing a helmet correctly (83.5%) decreased significantly from the estimated pre-campaign percentage. This overall percentage is also far lower than the WHO target of 100% by 2030.^2^^,^^4^ Contrary to best practice,^3^^,^^6^ the enforcement component of the campaign does not require helmets to be properly fastened and therefore does not adequately incorporate public education concerning correct helmet use.^10^^,^^11^^,^^13^ A phenomenon similar to our observation was reported in Viet Nam, where an increase in correct helmet wearing was not observed despite substantial increases in helmet wearing following the 2007 implementation of a new policy. Viet Nam revised its national policy in 2008 to address this challenge, after which incorrect helmet use was observed to diminish.^27^^,^^28^

The decreasing trend in correct helmet use may also reflect the fact that new users of helmets may have had no knowledge of or interest in the correct wearing of their helmet. Helmets may have been worn simply to meet legal requirements and not for personal safety, a possibility supported by the evidence that the presence of visible police enforcement was associated with increased helmet use among motorcyclists.^29^ The variations in estimated percentages of correct helmet wearing between groups may be associated with differences in both personal risk perception^30^ and external interventions such as on-site motorist counselling programmes.^26^^,^^31^

Our study had several limitations. First, our observations were limited to one city so generalizability to other locations, particularly rural areas, should be assessed through future research. Second, our study only assessed the short-term effectiveness of the national helmet promotion campaign, about one year after its implementation; the longer-term impact might decrease over time and should be evaluated. Third, viewer judgement during manual transcription of data from film, in terms of riding behaviour and rider characteristics, may have resulted in bias. The viewers were unaware of the study hypotheses and did not participate in statistical analyses, but they did know which round of observations (i.e. pre- or post-campaign) they were coding. Correct helmet wearing was coded based on an imperfect proxy technique from previous research,^17^^,^^18^ that is, whether the chinstrap was fastened tightly or else unfastened or fastened loosely. Both sex and age were judged inexactly based on physical appearance. Despite these challenges, our pilot research demonstrated a high accuracy in recording sex (99.7%; 304/305) and age group (93.1%; 284/305), and the reproducibility coefficients of over 10 hours of the 192 hours of film exceeded 92.0% for the studied variables.^15^ These results suggest that bias from manual transcription was unlikely to substantially change our results. Fourth, because components of the campaign were conducted in a mixed and comingled manner, we could not assess the effectiveness of each component separately. 

Our findings have two implications for policy. First, we determined that the multiapproach national helmet promotion campaign was successful and should be maintained, but some aspects of the campaign could be improved to yield a stronger effect. We recommend extending the campaign to cover all riders, including traditional cyclists, rather than focusing on e-bike riders and motorcyclists. According to best-practice recommendations,^6^ a requirement not just to wear a helmet but for the helmet to be properly fastened should be considered in legislation and enforcement. We also recommend incorporating correct helmet use into both the goals of the campaign and public education, as well as expanding the administration of on-site motorist counselling programmes to all cyclists and motorcyclists not wearing helmets. One challenge to the implementation of such campaigns is balancing the behavioural components with enforcement of policy. Research suggests that the enforcement of legal requirements is likely to yield more benefit in terms of improved road safety than simply behavioural and/or educational interventions on their own,^6^^,^^8^^,^^9^ but a combination of legal enforcement and education is likely to be most effective. For maximum benefit, carefully developed, theory-driven behavioural and educational interventions should be implemented along with the creation and then enforcement of legal policies.

A second implication of our results is that long-term mechanisms should be established by the government to ensure required resources are available to implement the campaign nationwide in a sustainable and continuous way. Government support or philanthropic donations will continue to be required to implement the intervention broadly, including the distribution of helmets to those who need them in underdeveloped and rural locations. Sporadic and scattered campaign implementation is unlikely to yield lasting benefit.

Although a substantial increase on pre-campaign helmet wearing, our estimated percentage of riders wearing helmets post-campaign is lower than that reported by WHO in high-income countries and in some low- and middle-income countries such as Viet Nam (81.0% in 2013 for motorcyclists).^4^ This difference suggests that, despite the success of the campaign in China, it may remain inadequate to increase helmet use to the level observed elsewhere. We recommend revising the current campaign to include all riders (i.e. traditional cyclists also) and to include education and legislation on the correct fastening of helmet chinstraps. Once proven effective, China’s campaign might be modelled by other countries to improve helmet use and accelerate progress towards the achievement of sustainable development goal target 3.6 for road safety.^32^
